# A Survey of Chinese Medicinal Herbal Treatment for Chemotherapy-Induced Oral Mucositis

**DOI:** 10.1155/2013/284959

**Published:** 2013-10-28

**Authors:** Gesa Meyer-Hamme, Kathrin Beckmann, Janine Radtke, Thomas Efferth, Henry Johannes Greten, Matthias Rostock, Sven Schröder

**Affiliations:** ^1^HanseMerkur Center for Traditional Chinese Medicine at the University Medical Center Hamburg-Eppendorf, Martinistraße 52, 20246 Hamburg, Germany; ^2^Department of Pharmaceutical Biology, Institute of Pharmacy and Biochemistry, Johannes Gutenberg University, Staudinger Weg 5, 55128 Mainz, Germany; ^3^ICBAS, University of Porto, Rua de Jorge Viterbo Ferreira No. 228, 4050-313 Porto, Portugal; ^4^Institute of Complementary Medicine, University Hospital Zurich, 8006 Zurich, Switzerland; ^5^Hubertus Wald Tumorzentrum, University Cancer Center Hamburg, University Medical Center Hamburg-Eppendorf, Martinistraße 52, 20246 Hamburg, Germany

## Abstract

Oral mucositis is one of the common side effects of chemotherapy treatment with potentially severe implications. Despite several treatment approaches by conventional and complementary western medicine, the therapeutic outcome is often not satisfactory. Traditional Chinese Medicine (TCM) offers empirical herbal formulas for the treatment of oral ulceration which are used in adaptation to chemotherapy-induced mucositis. While standard concepts for TCM treatment do not exist and acceptance by conventional oncologists is still low, we conducted a review to examine the evidence of Chinese herbal treatment in oral mucositis. Eighteen relevant studies on 4 single herbs, 2 combinations of 2 herbs, and 11 multiherbal prescriptions involving 3 or more compounds were included. Corresponding molecular mechanisms were investigated. The knowledge about detailed herbal mechanisms, especially in multi-herbal prescriptions is still limited. The quality of clinical trials needs further improvement. Meta-analysis on the existent database is not possible but molecular findings on Chinese medicinal herbs indicate that further research is still promising for the treatment of chemotherapy-induced oral mucositis.

## 1. Introduction

Oral mucositis is one of the most common side effects of chemotherapy treatment with potentially severe implications. According to the American National Cancer Institute, ulcerative oral mucositis occurs in approximately 40% of patients receiving standard-dose chemotherapy [1]. Medical interventions are required in about 50% of these patients, including changes of medication or chemotherapy dose reduction. Severe mucositis symptoms occur in up to 80% in high dose chemotherapy treatments of leukaemia or in stem cell transplant regimens [1].

Mucosal damages may be induced for example, by antimetabolites such as methotrexate, 5-fluorouracil, anthracyclines such as doxorubicin and bleomycin, alkylating antineoplastic agents such as cyclophosphamide and busulfan, taxanes and the platinum complexes, including cisplatin and carboplatin [1, 2]. All of them may have possible toxic effects on rapidly dividing mucosal cells, partly related to drug secretion in the saliva. Saliva volume and consistence as well as the oral microbial flora may be altered, affecting the mucosal metabolism [3]. Several molecular mechanisms are involved in the pathogenesis of mucositis, such as oxidation and apoptosis mediated by nitric oxide (NO), cyclooxygenase (COX), protein kinases, cytokines, and nuclear factors [4]. The research field involves also genetic-based risk factors [5]. Epigenetic changes of DNA methylation are discussed as being responsible for inflammatorial precancerous conditions [6]. A cancer diagnosis itself may lead to posttraumatic stress disorder (PTSD), causing depression and anxiety as well as an increased level of biomarker expression, such as interleukin-6 (IL-6), tumor necrosis factor-*α* (TNF-*α*), cortisol, and high-reactive sensitive C-reactive protein. The incidence of oral ulceration was associated with the level of PTSD. These effects were observed to be significantly higher in malignant than in benign diagnosed breast tumor patients [7].

Typical manifestations of oral mucositis are soreness, edema, erythema, ulcerations, bleeding, pain, difficulties in swallowing and possible alteration of taste, and they may severely affect the patient's quality of life. Impaired nutrition and complications by viral, bacterial, or mycotic infections may additionally increase the risk of anticancer treatment delay [8]. Mucositis grading is based on clinical aspects and the nutritional state, as seen in Table 1. Grades III and IV are considered as severe mucositis [9, 10] and in more than one-third of these patients the next chemotherapy cycle needs to be delayed leading to a possible deterioration of treatment [11].

When uncomplicated by infections, mucositis may be self-limiting in about 2 to 4 weeks [1]. Mucosal damages and local factors such as periodontitis and suboptimal oral hygiene increase the risk of infections. Systemic exacerbation is facilitated by the commonly decreased immunological status [12, 13]. Consequences are an increased mortality risk as well as a prolonged hospitalisation, including the necessity of fluid replacement and parenteral nutrition, causing an increase of costs [9].

### 1.1. Preventive and Treatment Methods by Conventional Medicine

The detailed guidelines for prevention and treatment of mucositis are depending on the chemotherapy regimens used in each case. General prevention instructions include prior dental examinations and treatment and optimal oral care [10, 16], as well as avoidance of spicy, hard and hot foods and saline-peroxide mouthwashes [16]. 

There are numerous western experimental preventive and therapeutical interventions for oral mucositis. Updated clinical practice guidelines for the prevention and treatment of mucositis were published by the Mucositis Study Section of the Multinational Association of Supportive Care in Cancer and the International Society for Oral Oncology (MASCC/ISOO) in 2007 [10], suggesting the use of keratinocyte growth factor-1 (KGF-1) for preventing mucositis in high-dose chemotherapy regimens. Cryotherapy was suggested for melphalan, 5-fluorouracil, and etidronate. Systemic glutamine was not recommended because of severe toxicity. Mouthwashing with granulocyte-macrophage-colony stimulating factor (GM-CSF) did not show consistent effect. Updated results are frequently published by MASCC/ISOO [17]. According to a Cochrane review from 2010, nine interventions for prevention and treatment of mucositis showed statistical benefit: allopurinol, amifostine, cryotherapy, intravenous glutamine, honey, KGF-1, laser, aloe vera, and polymixin/tobramycin/amphotericin (PTA) antibiotic pastille or paste compared to either placebo or no treatment [18]. The updated Cochrane review, published in February 2013, came to the conclusion that cryotherapy and keratinocyte growth factor had some benefits in preventing mucositis and sucralfate showed effects in reducing the severity of mucositis. Aloe vera, amifostine, granulocyte growth factor, honey, laser, and PTA did not show consistent effects [19]. 

Even concerning the progress achieved during the last years, chemotherapy-induced oral mucositis continues to be a challenge for a positive cancer treatment outcome [2, 18, 19]. The development of further treatment options for oral mucositis remains an important research objective. 

### 1.2. Complementary Medicine with Western Herbs

In western complementary medicine several herbal treatment approaches are existent, including *Salvia officinalis, Camomilla matriciana, Calendula officinalis, Hamamelis virginiana, Tormentilla rhizome, Commiphora molmol, Rhataniae radix, Myrtilli fructus, Althaea, Malva, Cetraria islandica, Linum usitatissimum, Caryophylli flos, Hippophea rhamnoides, Aloe vera, Carica papaya, Centaurii herba, Gentianae radix, Menyanthis folium, Eriodictyon crassifolium, Oleum olivae, and Citrus limon*. They are applied as single infusions for gargling or topical application [2]. Of these, Salvia officinalis, Chammomilla matriciana, Aloe vera, and Gentianae radix have also been used in the tradition of TCM.

Up to 80% of cancer patients use some kinds of Complementary and Alternative Medicine (CAM) therapies to support their conventional cancer treatments [20, 21]. Herbal treatment is the most frequently used CAM therapy and many of the used herbs originate from TCM [22, 23]. TCM offers empirical herbal formulas for treating mouth ulcers and stomatitis which have frequently been used in complementary treatment of oral mucositis in the last decades, but the evidence of these therapies is unclear. While standard concepts for this kind of treatment do not exist and acceptance by conventional oncologists is still low, we conducted this review to critically examine the evidence of Chinese herbal treatment in oral mucositis.

## 2. Methods

### 2.1. Objective

The objective of this article is to examine the role of Chinese herbal medicine approaches to oral mucositis in search of adjuvant treatment options for minimizing a painful and risky side effect of chemotherapy as a potential cooperation of western and Chinese medicine.

### 2.2. Search Strategy and Selection Criteria

Electronic searches of PubMed, MEDLINE via OVID, EMBASE via OVID, Cochrane Database, CNKI and reference lists of relevant articles were undertaken. The mesh-terms used were chemotherapy, chemotherapy-induced, oral ulcer, mouth ulcer, oral mucositis, stomatitis, Chinese herbal medicine, medicinal herbs, Chinese herbs, traditional Chinese medicine, antioxidant, anti-inflammatory. All interventional clinical trials concerning chemotherapy-induced oral mucositis treated by Chinese herbal medicine that offered an English or Chinese abstract were reviewed. Articles investigating herbal or animal products used in the tradition of Chinese medicine were included. Articles investigating radiochemotherapy-induced oral mucositis were excluded, unless the chemotherapy-related results were separately processed.

### 2.3. Data Analysis

No meta-analysis was effected since there were reservations with regard to the high risk of bias due to inadequate study designs and a diversity of herbal formulas. We limited the discussion to the comparison of single herbs and herbal formulas and to the quality of studies which have to date been set up with regard to this topic. 

## 3. Results

A total of 686 articles were retrieved from electronic searches and from examination of reference lists of clinical and review articles. After screening titles and/or abstracts, 632 articles were excluded since the focus was either on an intervention rather than on oral mucositis and Chinese herbal treatment or they were duplicated studies or not relevant. From a total of 54 articles which were retrieved for detailed evaluation, 18 studies representing 1,476 patients met the selection criteria and were included in the review, focusing on 4 single herbs, 2 combinations of 2 herbs, and 11 multi-herbal prescriptions involving 3 or more components. For a summary of the investigated studies, see Table 2.

## 4. Single Herbs or Single Herbal Compounds

### 4.1. *Evodiae fructus*

In terms of TCM theory, *Evodiae fructus* has been used for nausea and pain caused by cold exposition [24–26]. Evodiamine, a major compound of *Evodiae fructus*, was found to inhibit inducible nitric oxide synthase (iNOS) and nuclear factor kappa-light-chain-enhancer of activated B cells (NF-*κ*B) activation, as well as the cyclo-oxygenase-2 (COX-2) expression, hypoxia-inducible factor 1-alpha (HIF-1-*α*) accumulation and prostaglandin E-2 (PGE-2) synthesis, and interferon-gamma (INF-*γ*) mediated processes in murine 264.7 macrophage-like cell lines [27].

Application of *Evodiae fructus* extract on acupoint Yongquan (KID1) was reported to have positive effects on mucositis symptoms in 92% of chemotherapy patients in an uncontrolled case study (*n* = 50) measured by subjective scales [28]. Ulcer grading has not been mentioned [28]. The result requires further investigation with higher-quality study designs.

### 4.2. *Rhodiola algida*

*Rhodiola* subspecies are used as common tonics in various Asian regions. In Tibetan medicine, it has an ancient tradition for its antifatigue effects, as well as for cardiovascular diseases, pneumonia, and hemoptysis [29]. *Rhodiola algida* is externally used for injuries, burns, and scalds [29] and has shown immunomodulatory effects by interleukin 2 (IL-2) regulation in Th-1 cells and interleukin 4, 6, and 10 (IL-4, IL-6, IL-10) regulation in Th-2 cells [30]. In a randomised, controlled, two-armed clinical study on breast cancer patients (*n* = 130; treatment: *n* = 65; control: *n* = 65) [31], oral administration of *Rhodiola algida* resulted in faster recovery of white blood cell count as well as fewer and smaller ulcers compared to the non-treatment control group, judged by clinical measurement. Furthermore, positive effects of *Rhodiola algida* on lymphocyte proliferation was reported while animal feeding with *Rhodiola algida* did not show toxic effects [31]. These findings are promising, but so far no further trials confirming the results have been found. 

### 4.3. *Catechu* from *Acacia catechu*

*Catechu* is an extract of *Acacia catechu *which is clinically used for tissue regeneration, wound healing, sores and abscesses as well as mouthwash for oral ulcers [26, 32]. In combination with *Scutellariae baicalensis radix*, anti-inflammatory effects are reported in animal and human immortalized cell lines [33]. Local application of Catechu powder had a superior effect on oral mucositis compared to local norfloxacin application in a randomized, controlled, two-armed clinical study on chemotherapy patients (*n* = 60; treatment: *n* = 30; control: *n* = 30), judged by clinical measurement [34]. Amelioration occurred in all patients (100%) treated with *Catechu *and in 73.3% of the norfloxacin group. Further trials confirming this result have not been found.

### 4.4. Kangfuxin from *Periplaneta americana*

Kangfuxin is an ethanolic extract of *Periplaneta americana*, used for its anti-inflammatory and wound healing qualities in ulcerative and inflammatory diseases, including recurrent aphthous ulcers [35]. Regulating effects of the cytokine expression IL-4, interleukin 5 (IL-5), IFN-*γ*, and TNF-*α* by decreased gene expression have been reported [36]. In a randomized, controlled, two-armed clinical study on chemotherapy patients (*n* = 64; treatment: *n* = 26; control: *n* = 38), topical Kangfuxin application showed an oral mucositis rate of 36%, compared to saline gargling with a mucositis rate of 84% [37]. This result as well requires verification by more trials with high-quality study designs.

## 5. Herbal Combinations

### 5.1. *Lonicerae flos* Plus *Glycyrrhizae radix*

*Lonicerae flos* and *Glycyrrhizae radix* are two important ingredients of the traditional herbal formula *Yin Qiao San (Chinese) (Honeysuckle and Forsythiae Powder (English), Pulvis Lonicerae et Forsythiae (Latin))* from *Wen Bing Tiao Bian* (Systematic Differentiation of Warm Diseases) [38]. It is used in inflammatory conditions including those of skin and mucosa [39, 40]. In TCM tradition, *Lonicerae flos* is used for abscesses, swelling, ulcers and erysipelas [24–26]. It offers antioxidant properties, suppressing interleukin 1-beta (IL-1*β*), IL-6, and COX-2 gene expression in human umbilical vein cells [41]. In traditional treatment, *Glycyrrhizae radix* is known to be useful for abscesses, scores, and ulcer treatment [24–26]. Anti-inflammatory effects are transmitted by variant compounds. Glycerol inhibits lipopolysaccharide (LPS)-induced NF-*κ*B, IL-1, and IL-6 mRNA activation, while liquiritigenin was found to inhibit the activation of NF-*κ*B in macrophages, decreasing iNOS and proinflammatory cytokines by inhibition of LPS-induced NF-*κ*B DNA binding activity [42, 43].

This herbal combination has been investigated by two authors. In one randomized, controlled, two-armed clinical trial, the combined topical and internal use was compared to borax mouth washing in chemotherapy patients (*n* = 190; treatment: *n* = 97; control: *n* = 93). Mucositis symptoms were found in 19.4% of treatment group patients and in 85.2% of control group patients, measured by the WHO scale for mucositis [44]. The study did not establish a control for the internal use of herbal medicine, comparing a dual-mode treatment to a single control. In another clinical trial on chemotherapy patients (*n* = 86; treatment: *n* = 43; control: *n* = 43), herbal gargling solution was compared to hydrogen peroxide mouth washing [45]. Positive effects on mucositis symptoms were found in 95.3% of the herbal treatment group and 76.7% of the control group, measured by a subjective scale. Further investigation by high-quality studies is required.

### 5.2. *Lonicerae flos* Plus *Glycyrrhizae radix* Plus *Astragali membranacei radix*

In this formula, *Astragali membranacei radix* is used in addition to the herbs described above, *Lonicerae flos* and *Glycyrrhizae radix*. In traditional use, *Astragali membranacei radix* clears toxicity and is used for abscesses and promoting skin regeneration [24–26]. It has anti-inflammatory and immunoregulatory effects due to IL-2 and IFN-*γ* release and IL-4 and iNOS suppression [46].

Topical use of this triple combination on oral mucositis was effected in a randomized, controlled, two-armed clinical trial (*n* = 97; treatment: *n* = 50; control: *n* = 47), with positive results compared to *Dobell's solution* (sodium borate, sodium bicarbonate, phenol and glycerol) [47]. The herbal treatment group showed an overall lower grade of ulcers. 30% did not show any mucositis symptoms while only 2% suffered from severe ulcers. In the *Dobell's solution* control group, 5% had remained without ulcers while 21.3% suffered from severe ulcers. There was no statistical difference in the duration of healing period. In addition to the investigated drugs, all patients received a basic treatment by intravenous injections of the Chinese herbal preparations *Shengmai* (*Ginseng radix, Ophiopogonis radix, Schisandrae chinensis fructus*), *Shenqi Fuzheng* (*Codonopsis radix, Astragali membranacei radix*), *Ai Di* (*Acanthopanacis radix, Astragali membranacei radix, Ginseng radix*) and *Cantharidin*, as well as vitamin supplements not further described, and tetracaine solution for local application. In total, this is a strongly tonifying herbal regime. Regarding this treatment by applying several intravenously administered herbs, significant conclusions on the investigated herbs of the gargle solution cannot be drawn due to various potential interactions between the single components. 

### 5.3. *Lonicerae flos* Plus *Ophiopogonis radix* Plus *Platycodonis radix*

*Lonicerae flos* and *Ophiopogonis radix* are part of the classical herbal combination called *Qing Ying Tang (Chinese), Clear the Ying Level Decoction (English), Decoctum Refrigerationis Qi Constructivi (Latin)* from *Wen Bing Tiao Bian* (Systematic Differentiation of Warm Diseases) [38], which is composed by *Rhinoceri cornu, Rehmanniae viridae radix, Scrophulariae radix, Lophatheri folium, Lonicerae flos, Forsythiae fructus, Coptidis rhizoma, Salviae miltiorrhizae radix* and *Ophiopogonis radix*. It is used for certain forms of fever accompanied by a dry mouth [39, 40]. *Ophiopogonis radix* and *Playcodonis radix* are combined in *Bai He Gu Jin Tang (Chinese), Lily Bulb Decoction To Preserve The Metal (English), Decoctum Firmans Metallum cum Lilio (Latin)* from *Yi Fang Yi Jie (Analytic Collection of Medical Formulas)* [57], used for dry pharyngitis and bronchitis [39, 40]. Both formulas have other principal herbs whose effects are assisted by *Lonicerae flos, Ophiopogonis radix* and/or *Platycodonis radix* [39, 40]. 

In TCM theory,* Ophiopogonis radix* is used for sore throats and dry coughs [24–26]. There are scarce reports about single molecular mechanisms of *Ophiopogonis radix*. Opaw-2, a compound from *Ophiopogonis radix*, showed dose-dependent stimulation of lymphocyte proliferation in vitro [58]. *Platycodonis radix* had been traditionally used for relieving soreness, expelling pus and for treating general skin and mucosal diseases [24–26]. *Platycodonis radix* Saponins derived from *Platycodonis radix* showed anti-inflammatory effects via inhibition of COX-2, TNF-*α*, and PGE2 expression, as well as reduction of inflammatory markers like the number of leukocytes and neutrophiles and edema [59]. 

For *Lonicerae flos*, see above.

This triple combination was investigated in a randomised, controlled, two-armed clinical trial on chemotherapy patients (*n* = 65; treatment: *n* = 30; control: *n* = 35). The treatment group patients received oral herbal administration while the control group patients used furacilin mouth washing and received oral administration of vitamins B1 and C and daily intravenous infusions of metronidazol. Basic treatment was performed by saline gargling accompanied by diet instructions. The herbal treatment group showed improvement of ulceration in 93%, compared to 73.8% in the control group, classified by subjective scales of ulcer size [48]. In this trial, diversified control interventions impede the comparability to the treatment intervention. 

### 5.4. *Lonicerae flos* Plus *Glycyrrhizae radix* Plus *Chrysanthemi flos* Plus *Ganoderma lucidum* Plus *Menthae haplocalycis herba*

A *Lonicerae flos*, *Glycyrrhizae radix* and *Menthae haplocalycis herba* combination is found in *Yin Qiao San* [39, 40] as described above. A *Chrysanthemi flos*, *Glycyrrhizae radix* and *Menthae haplocalycis herba* combination is found in *Sang Ju Yin (Chinese), Clear Wind Heat Tea (English)i Potio Mori et Chrysanthemi (Latin)* from *Wen Bing Tiao Bian* [38] which contains *Mori folium*, *Chrysanthemi flos*, *Armeniacae semen*, *Forsythiae fructus*, *Menthae haplocalycis herba*, *Platycodi radix*, *Glycyrrhizae radix*, and *Phragmitis rhizome* and is used in beginning fever and coughing [39, 40]. *Ganoderma lucidum* has not been found in these classical combinations. 

In TCM theory, *Chrysanthemi flos* is used for septic wounds and abscesses [24–26]. *Chrysanthemi flos* compounds inhibit NO, PGE-2, TNF-*α*, and IL-1*β* production, as well as iNOS and COX-2 expression in LPS-induced macrophages [60]. *Ganoderma lucidum* showed positive effects on intestinal epithelium healing [61] and refractory diabetic wounds [62] and was found to decrease NO, PGE-2, and proinflammatory cytokine production, including IL-1*β*, TNF-*α* and NF-*κ*B in microglia [63]. *Menthae haplocalycis herba* in TCM theory is used for mouth sores, exanthema, and itching [24–26]. It showed antimicrobial activity against streptococcus mutans [64] and attenuated histamine release and PGD-2 synthesis in mast cells [65].

For *Lonicerae flos* and *Glycyrrhizae radix*, see above.

The decoction of *Lonicerae flos, Glycyrrhizae radix, Ganoderma lucidum, Chrysanthemi flos*, and *Menthae haplocalycis herba* was prepared for local ice cube application and compared to ambient tempered *Dobell's gargle solution* (sodium borate, sodium bicarbonate, phenol, and glycerol) in a randomised, controlled, two-armed clinical trial on gynaecological tumor patients receiving 5-FU chemotherapy (*n* = 217; treatment: *n* = 84; control: *n* = 133) [49]. The mucositis incidence resulted in 13.1% in the herbal treatment group and in 39.1% in the control group. The study design did not consider the fact that single ice application is known to have a preventive effect on oral mucositis in patients treated with 5-FU [1, 66], as there was no similar application design for both groups.

### 5.5. *Shengmai San* (Chinese), Generate the Pulse Powder (English)

*Shengmai San* contains *Ginseng radix, Ophiopogonis radix,* and *Schisandrae chinensis fructus *and originates from *Yu Xue Qi Yuan (Expounding on the Origins of Medicine)* [67]. It is used in multiple clinical patterns including cardiovascular and neurologic disorders, diabetes, and cancer for its tonifying and yin nourishing properties [39, 40]. Regarding the single compounds, there are multiple reports about anti-inflammatory effects of ginsenosides by inhibition of proinflammatory cytokines and other mediators of inflammation including iNOS, NO, IF-*γ*, COX-2, NF-*κ*B, and TNF-*α* [68]. Ginsenoside Rd showed wound healing effects on skin level, increasing the proliferation and migration of keratocyte progenitor cells and dermal fibroblasts by cyclic adenosine monophosphate (cAMP) induction via 9-*β*-d-arabinofuranoside attenuation [69]. *Schisandrae chinensis fructus* in terms of TCM theory is astringent and preserving fluids [24–26]. Schisandrin B was found to inhibit ataxia telangiectasia and Rad3-related (ATR) protein kinase activity following DNA damage by inhibition of phosphorylation processes [70]. A hydrophobic fraction of dried *Schisandrae chinensis fructus* was found to suppress IL-*β*-induced NO and iNOS expression, as well as the transcription of IL-1*β* and inflammatory cytokines [71]. 

For *Ophiopogonis radix*, see above.

In a randomised, controlled, two-armed clinical trial (*n* = 71; treatment: *n* = 36; control: *n* = 35) on acute chemotherapy toxicity regarding primarily white blood cell and platelet counts as well as nausea, vomiting, and oral mucositis, intravenous *Shengmai* injection was compared to dexamethasone, metoclopramide, and ondansetron injection (which targeted nausea and vomiting rather than mucositis due to the ampler study design). Oral mucositis occurred in 5.5% of the treatment group and in 28.5% of the control group [50]. The control intervention of this trial was not specific for oral mucositis.

### 5.6. *Bubali Cornu* and *Callicarpae macrophyllae folium*

*Bubali cornu *is frequently found as modern substitute for *Rhinoceri cornu* (forbidden due to the Convention on International Trade in Endangered Species of Wild Fauna and Flora, also known as Washington Convention) in the classical formula *Qing Ying Tang* (see above). There are no classical formulas combining these two drugs, but this is a modern empirical combination called *Shui Zhong Cao Tang Ji (Chinese), Water Grass Decoction (English)*. In terms of TCM theory, *Bubali cornu* has effects similar to *Rhinoceri cornu*, used for febrile diseases, exanthema, and convulsions [24–26]. It has shown antipyretic and antioxidant effects on proteins and inhibition of TNF-*α*-induced PGE2 production, as well as protection against hydrogen peroxide (H_2_O_2_)-induced injuries in rat cerebral microvascular endothelial cells [72]. *Callicarpae macrophyllae folium* is reported to be used in TCM tradition for bleedings, hematoptysis, and hematemesis [26]. Anti-inflammatory, antimicrobial, and analgesic effects of *Callicarpae macrophyllae folium* are reported but not yet fully investigated [73]. 

This formula was topically and internally administered in a randomised, controlled, three-armed clinical trial (*n* = 88; treatment: *n* = 30; first control: *n* = 30; second control: *n* = 28) to chemotherapy patients, compared to topical application of gentamycin, tetrahydropholate, and saline gargling (first control) and to oral oryzanol administration (second control) [51]. There was a mean better outcome of curative voted cases from herbal treatment to the gentamycin/tetrahydropholate/saline control group (53.3% to 50.0%). General improvement of oral mucositis symptoms was seen in 93.3% of both groups. Oral mucositis symptoms remained in 92.2% of the oryzanol group, showing an advantage of the two former regimens compared to oxyzanol administration alone. The comparison of the combined topical and internal use to a single control intervention does not allow significant conclusions. 

### 5.7. *Huang Wu Shu Kou Ye* (Chinese), Yellow Five Decoction (English) (*Phellodendri Chinensis Cortex*, *Forsythiae fructus*, *Verbenae officinalis herba*, *Borneolum*, *Galla chinensis*, and *Catechu*)

The name of this empirical prescription may be borrowed from *Dang Gui Liu Huang Tang, Angelica Six Yellows Decoction (English), Decoctum Angelicae Sinensis et sex Luteorum (Latin)* from *Lan Shi Mi Cang* (Sectrets from the Orchid Chamber) [74], as *Phellodendri chinensis cortex* is one of the contained 6 yellow coloured drugs (*Rehmanniae viridae radix, Rehmanniae preparata radix, Coptidis rhizoma, Scutellariae baicalensis radix, Phellodendri chinensis cortex, and Astragali membranacei radix*). The combination of *Phellodendri chinensis cortex, Forsythiae fructus, Verbenae officinalis herba, Borneolum, Galla chinensis*, and *Catechu* has not been found in classical prescriptions.

*Phellodendri chinensis cortex* is traditionally considered as anti-toxic and is used for abscesses and sores [24–26]. It was found to inhibit TNF-*α*, IL-1*β*, and iNOS production, as well as phosphorylation of extracellular-signal regulated kinases (ERK) and NF-*κ*B activation in microglia cells [75]. *Forsythiae fructus* has a traditional use as anti-toxin as well as for erysipelas and abscesses [25–27]. A *Forsythiae fructus* compound, arctiin was found to decrease proinflammatory cytokine production including IL-1*β*, IL-6, TNF-*α*, and PGE-2, as well as NF-*κ*B and co-stimulating molecules such as peripheral membrane protein B7-1 and B7-2 in mouse leukaemic monocyte macrophage cell line (RAW 264.7) cells [76]. *Verbenae officinalis herba* in TCM terms removes toxicity and is used for sores and boils as well as pharyngitis [26, 77]. *Verbenae officinalis herba* extractions were found to possess antioxidant, anti-inflammatory, and wound-healing properties [78]. Borneolum is a crystal steam distilled product of *Cinnamomum camphora* [26]. It is used externally for mouth sores, ulcerations, and wounds [24–26] and showed anti-inflammatory and antioxidant cell protective effects by decreasing iNOS expression, NO, and inflammatory factor release, as well as NF-*κ*B translocation and caspase-related apoptosis in an ischemic/reperfusion neuron model [79]. *Galla chinensis* in TCM theory astringes, promotes wound healing and is used for ulcers and edema [24–26].

For *Catechu*, see above.

A gargle solution composed by these 5 herbs as well as saline gargle solution was administered in a randomised, controlled, two-armed clinical trial (*n* = 101; treatment: *n* = 53; control: *n* = 48) to chemotherapy patients, compared to *Borax* solution gargling alone. All patients received basic treatment with antibiotics and vitamin supplements not further described. Improvement on mucositis symptoms was seen in 96.2% of the treatment group and 76.1% of the control group, judged by subjective clinical scales [52]. Significant conclusions on this result cannot be drawn as antibiotic regimens may have interfered with possible anti-inflammatory effects of the herbal solution used at the same time. 

### 5.8. *Yu Nu Jian* (Chinese), Jade Woman Decoction (English)

This classical TCM formula containing *Gypsum fibrosum*, *Rehmanniae viridae radix*, *Anemarrhenae rhizoma*, *Ophiopogonis radix*, and *Achyranthis bidentatae radix*, is described in *Collected Treatises of Zhang Jing Yue* [80]. It is used for inflammatory diseases including oral ulcerations [39, 40]. *Gypsum fibrosum* is traditionally used for burns and ulcers [24, 25] as well as for fever [24–26], and it showed antipyretic activity demonstrated against LPS-induced pyrexia in rats, while calcined Gypsum and CaSO_4_ did not have this effect [81]. *Rehmanniae viridae radix* had been traditionally used for exanthema, abscesses, and sore throats [24–26]. Compounds of *Rehmanniae viridae radix* were found to inhibit NO production and iNOS, PGE-2, IL-6, and COX-2 expression in RAW 264.7 macrophages [82]. *Anemarrhenae rhizoma* had been traditionally used for dry coughs and infectious diseases [24–26]. Nyasol, a compound of *Anemarrhenae rhizoma*, was found to reduce NO and PGE-2 production as well as mRNA-levels of TNF-*α* and IL-1*β* in LPS-stimulated microglia cells. P38 mitogen-activated protein kinase (p38MAPK) was inactivated and LPS-induced I-*κ*B*α* degradation was suppressed [83]. *Achyranthis bidentatae radix* calms bleeding from the mucosa in TCM theory and is used for mouth soreness, epistaxis, and hematemesis [24–26]. *Achyranthis bidentatae* polysaccharides derived from *Achyranthis* were found to positively modulate murine dendritic cell maturation by cell surface molecules CD86 and CD40 and major histocompatibility complex II (MHC II) enhancement and increase IL-12 production, indicating a possible immune boosting effect [84].

As *Yu Nu Jian* was investigated with *Qing Wei San (see below)* in one study, oral mucositis related results are stated below.

### 5.9. *Qing Wei San* (Chinese), Clear the Stomach Powder (English)

This classical TCM formula contains *Coptidis rhizoma*, *Cimicifugae rhizoma, Rehmanniae viridae radix, Moutan cortex, Angelicae sinensis radix*, and *Achyranthis bidentatae radix*. It is described in *Lan Shi Mi Cang* (Secrets from the Orchid Chamber) [74] and used for gingivitis and inflammations of tongue and lips.

In terms of TCM theory, *Coptidis rhizoma *is regularly used in inflammatory and septic processes [24–26]. It was found to inhibit IL-1*α*, IL-6, and granulocyte macrophage colony-stimulating factor (GM-CSF) secretion, iNOS expression, and NO production in RAW 264.7 macrophages [85]. *Cimicifugae rhizoma* in TCM terms removes toxicity and is used in exanthema, mucosal inflammation, and ulceration [24–26]. It was found to reduce LPS-induced release of IL-6, TNF-*α*, IFN-*γ*, and stimulation of IL-8 in LPS-induces human blood cells [86]. *Moutan cortex* in TCM terms is used in cases of inflammation and mucosal bleeding [24–26]. It was found to inhibit the activation of several inflammation-related genes in gingival fibroblasts [87]. *Angelica sinensis radix* is used for sores, ulcers, and abscesses in TCM theory. Ligustilide, an *Angelica sinensis radix* compound, was found to suppress NO production, PGE-2, and TNF-*α* in LPS-stimulated RAW 264.7 macrophages, to decrease activator protein-1 (AP-1), iNOS and NF-*κ*B activation, phosphorylation of I*κ*B kinase (IKK), MPAKs, ERK1/2, and c-Jun-N-terminale kinase (JNK) and to downwardly regulate intracellular reactive oxygen species (iROS) [88]. 

For *Rehmanniae viridae radix* and *Achyranthis bidentatae radix*, see above.

Both *Yu Nu Jian (see above) *and *Qing Wei San* were investigated in an uncontrolled case study on leukaemia patients (*n* = 31), clinically allocated into two groups according to the criteria of exuberance or deficiency by terms of TCM theory. *Yu Nu Jian *was administered to the exuberance group while *Qing Wei San* was administered to the deficiency group. Additional topical medication was effected in all patients without further description. Ulcer grading was clinically judged. Reported without group differentiation, 7 patients obtained very good results and 19 patients offered good results on mucositis symptoms, indicating an improvement of 83.9% in summary [53]. In the context of a scientific study, the simultaneous use of different formulas precluded significant results. The findings require further investigation by higher-quality trials.

### 5.10. *Chrysanthemi flos* Plus* Gardeniae fructus* Plus *Hyperici perforati herba* Plus *Scrophulariae radix* Plus *Sophorae tonkinensis radix* Combination

Classical combinations of these herbs have not been found. Obviously, the formula was empirically composed with the purpose of obtaining anti-inflammatory and wound-healing effects. In terms of TCM theory, *Gardeniae fructus* had traditionally been externally used for wounds and contusions [24–26]. Geniposide from *Gardeniae fructus* were found to inhibit TNF-*α*, IL-6, and IL-1*β*, to block the phosphorylation of I*κ*B*α* and transcription factor p65 and p38, as well as extracellular-signal-regulated kinases (ERK) and c-Jun N-terminal kinases (JNK), and to decrease the toll-like receptor-4 (TLR4) expression in LPS-simulated macrophages. They were also found to decrease the LPS-induced IL-8 production in human embryonic kidney cells (HEK293-mTLR4/MD-2 cells) [89]. *Hyperici perforati herba* is astringent and expels toxins in TCM theory [26]. *Hyperici perforati herba* compounds were found to inhibit LPS-induced PGE-2, COX-2, and NO through the suppression of cytokine signaling 3 (SOCS3) activation in 264.7 macrophages [90]. *Scrophulariae radix* in TCM theory is classified as removing toxicity and moistening tissues; it is used for sores and exanthema [24–26]. *Sophorae tonkinensis radix* had been traditionally used for tonsillitis, pharyngitis, and mouth sores [26, 91]. It is toxic in higher doses. In vitro, it showed antiviral activity on Coxsackie-, Echo-, and Polio-virus [92].

For *Chrysanthemi flos*, see above.

As this prescription was investigated in one study with Qing Dai San (see below), mucositis related results are stated below.

### 5.11. *Qing Dai San*

*Borax *and *Borneolum* combination is found in *Bing Peng San (Chinese), Borneol and Borax Powder (English), Pulvis Borneoli et Boracis (Latin)*, as a locally used prescription for oral inflammation and aphthous ulceration from *Yi Zong Jin Jian* (Golden Mirror of the Medical Tradition) [93]. An alternative to this prescription, *Qing Dai San* contains *Borax*, *Borneolum*, and *Indigo naturalis* [39, 40]. *Borax* is an extract of natural borax mineral. In terms of TCM theory, it removes toxicity from skin and mucosa upon external application [24, 25]. Borax compounds were found to reduce genotoxic effects of heavy metal exposure of human blood cell cultures by arsenic, bismuth, cadmium, mercury, and lead, normalising decreased antioxidant enzyme activities as well as sister chromatid exchange and micronuclei and plasma malondialdehyde (MDA) levels [94]. High *Borax* doses showed toxic cellular effects, decreasing human lymphocyte proliferation and increasing sister chromatid exchange in chromosomes [95].

Indigo naturalis is the fermented and chalked extract of *Strobilenthes flaccidifolius, Indigo tinctoria, Isatis oblongata* or *Polygonaum tinctorium*. It is used for exanthema and ulcers in TCM [24–26] and was found to to inhibit superoxide anione generation as well as MPAK phosphorylation and calcium mobilisation in formyl-methionyl-leucyl-phenylalanine (FMLP)-activated human neutrophils [96].

For *Borneolum*, see above.

The compositions 5.10 and 5.11 were investigated together in a randomised, controlled, four-armed clinical trial on both chemotherapy- and radiation-induced oral mucositis. This article refers only to the two-armed part concerning chemotherapy patients (*n* = 60; treatment: *n* = 30; control: *n* = 30). The decoction of *Chrysanthemi flos, Gardeniae fructus, Hyperici perforate herba, Scrophulariae radix*, and* Sophorae tonkinensis radix* was orally administered to the treatment group who also used a gargle solution composed of *Borax*, *Borneolum*, and *Indigo naturalis*. The control group patients received vitamin B12 administration and used a gentamycin/sodium//bicarbonate gargle solution. Ulcer grading was defined by size and quantity of ulcers. Improvement on oral mucositis symptoms were found in 96.7% of the herbal treatment group, compared to 86.7% in the control group. An overall shorter healing period for the herbal treatment group was reported [54]. In this trial, the investigation of two formulas at the same time compared to disparate controls impeded clear results.

### 5.12. *Codonopsitis radix* Plus *Atractylodis macrocephalae Rhizome *Plus *Glycyrrhizae radix* Plus *Angelicae sinensis radix* Plus *Rehmanniae viridae radix* Plus *Astragali membranacei radix* Plus *Dioscoreae oppositae rhizoma* Plus *Alismatis rhizoma* Plus *Agastachis herba* Plus *Lophatheri herba*

This formula may be regarded as a modified incomplete *Ba Zhen Tang (Chinese)*, Eight Treasure Tea (English), Decoctum octo Gemmarum (Latin), from *Zhen Ti Lei Yao* (Catalogued Essentials for Correcting the Body) [97], which is a strong formula for devitalised patients, composed *by Ginseng radix* (or alternatively *Codonopsitis radix*), *Atractylodis macrocephalae rhizoma*, *Glycyrrhizae radix*, *Angelicae sinensis radix*, *Rehmanniae radix*, *Poria alba*, *Paeoniae alba radix,* and *Ligustici chuangxiong rhizoma* [39, 40]. In prescription 5.12, the last three herbs of Ba Zhen Tang were replaced by *Astragali membranacei radix, Dioscoreae oppositae radix, Alismatis rhizoma, Agastachis herba*, and *Lophatheri herba*, directing the prescription to immune consolidating effects [24–26].

*Agastachis herba, Atractylodis macrocephalae radix,* and *Glycyrrhizae radix* are found in *Huo Xiang Zheng Qi San* (Chinese), Agastache Powder to Rectify the Qi (English), *Pulvis Agastachis pro Qi Orthopathico (Latin)* from *Tai Ping Hui Min He Ji Ju Fang* (Imperial Grace Formulary of the Tai Ping Era) [98] with *Magnoliae cortex, Citri reticulatae pericarpium, Perillae folium, Angelica dahuricae radix, Pinelliae rhizoma, Arecae pericarpium, Poria alba,* and *Platycodonis radix*. It is used for endemic infections and gastritis [39, 40]. *Alismatis rhizoma, Codonopsitis radix, Atractylodis macrocephalae rhizoma, Angelicae sinensis radix, and Glycyrrhizae radix* are also found in *Dang Gui Nian Tong Tang (Chinese), Decoction to Lift the Pain (English) from Nei Wai Shang Bian Huo Lun* (Clearing Doubts about Injury from Internal and External Causes) [99] with *Atractylodis radix*, *Ledebouriellae radix, Puerariae radix, Scutellariae radix, Anemarrhenae rhizoma, Artemisiae herba, Polyporus, Sophorae radix, Notopterygii radix*, and *Cimicifugae radix*. It is used for inflammatory diseases such as arthritis, impetigo, and eczema [100]. 

*Codonopsitis radix* extract was found to inhibit NO, TNF-*α*, IL-3 IL-6, and the ERK signalling pathway as well as LPS-induced phagocytic uptake and CD29-mediated cell-cell-adhesion in RAW 264.7 macrophages [101]. *Dioscoreae oppositae rhizoma* was found to decrease the NO and proinflammatory cytokine production including IL-1*β*, Il-6, TNF-*α*, and PGE-2, as well as iNOS, and the COX-2, and NF-*κ*B activation in RAW 264.7 macrophages [102]. *Alismatis rhizoma *is diuretic in TCM theory [24–26]. It is not typically used for oral diseases but was found to suppress NF-*κ*B, COX-2, IL-1*β* and iNOS, as well as induced nuclear factor-like 2 (Nrf2)-regulated gene expression in RAW 264.7 cells [103]. *Agastachis herba* in TCM theory has antiedematous effects and is used for nausea and fever [24, 25]. *Agastachis herba* extract showed antioxidant effects increasing heme oxygenase-1 (HO-1) enzyme activity by way of the protein kinase G (PKG) signalling pathway in RAW 264.7 macrophages [104]. *Lophatheri herba* has been traditionally used for mouth and tongue sores [26]. Glycosides derived from *Lophatheri herba* were found to possess an anti-respiratory syncytial virus (RSV) effect in vitro [105]. 

For *Atractylodis macrocephalae rhizoma, Glycyrrhizae radix, Astragali membranacei radix, Angelicae sinensis radix,* and *Rehmanniae viridae radix*, see above. 

The decoction of *Codonopsitis radix, Atractylodis macrocephalae rhizoma, Glycyrrhizae radix, Angelicae sinensis radix, Rehmanniae viridae radix, Astragali membranacei radix, Dioscoreae oppositae rhizoma, Alismatis rhizoma, Agastachis herba,* and *Lophatheri herba* was administered in a randomised, controlled, two-armed clinical trial (*n* = 66; treatment: *n* = 33; control: *n* = 33) to chemotherapy patients during 4 chemotherapy cycles. The treatment group received not only the herbal decoction but also a multi vitamin, mineral, and micronutrient supplement (Centrum Wyeth). The control group patients received only vitamin B2 administration. Oral ulcer incidence grew from the first to the fourth chemotherapy cycle up to 21.2% in the treatment group and 66.7% in the control group, judged by WHO scale for Oral Mucositis. The increase was not only higher but also faster in the control group [55].

In this trial, the diversified vitamin supplement application confused the effect of the herbal medicinal treatment. Vitamin B2 is not a valuable control to any multivitamin supplement and/or Chinese medicinal herbs. Basic conditions should be equal in both groups in order to achieve a measurable effect. The effect of vitamin supplements should be investigated independently from herbal medicine.

### 5.13. Modified *Xie Huang San *(Chinese), Drain the Yellow Powder (English)

The classical prescription *Xie Huang San contains Gypsum fibrosum, Saposhnikoviae radix, Gardeniae fructus, Agastachis herba*, and *Glycyrrhizae radix*. It is described in *Xiao Er Yao Zheng Zhi Jue* (Craft of Medicines and Patterns for Children) [106] and used for inflammatory diseases of stomach and mouth [39, 40].

*Saposhnikoviae radix* is used for affections of skin and mucosa [24–26]. It was found to inhibit NO production through iNOS and its mRNA expression in LPS-induced RAW 264.7 cells [107]. 

For the further herbs of this formula, see above.

Modifying standard prescriptions by herb addition related to syndrome patterns or individual symptoms is common in TCM tradition [39, 40]. In a clinical trial on 90 chemotherapy patients [56], *Xie Huang San* was administered amended by addition of *Ginseng radix, Astragali membranacei radix, Atractylodis macrocephalae rhizoma, Coptidis rhizoma, Taraxaci herba, Dendrobii caulis, Hedyotidis herba*, and *Lophatheri herba* which offer additional anti-inflammatory qualities [24–26]. 

In terms of TCM theory, *Taraxaci herba* is used for swelling, abscesses, and sore throat [24–26]. Taraxacosterol a flavonoid, isolated from *Taraxaci herba*, was found to inhibit NO, PGE-2, TNF-*α*, IL-1*β*, and IL-6 production as well as LPS-induced NF-*κ*B translocation in RAW 264.7 macrophages [108]. *Dendrobii caulis* had been used for dry mouth in TCM theory [26]. Anti-inflammatory and saliva secretion increasing effects of *Dendrobii caulis* were indicated in a Sjögren's mouse model [109]. *Hedyotis herba* in TCM theory has been used for abscesses and ulcers [24, 25].

For *Ginseng radix, Atractylodis macrocephalae rhizoma, Coptidis rhizoma*, and *Lophatheri herba*, see above.

*Astragali membranacei radix, Atractylodis macrocephalae rhizome*, and *Ginseng radix* are found in *Bu Zhong Yi Qi Tang* (Chinese), *Tonify the Middle and Augment the Qi Decoction (English), Decoctum Suppleens Centrum et Augmentans Qi (Latin)* from *Nei Wai Shang Bian Huo Lun* (Clearing Doubts about Injury from Internal and External Causes) [99] with *Glycyrrhizae radix tosta, Angelicae sinensis radix, Aurantii pericarpium, Cimicifugae rhizoma, and Bupleuri radix.* Furthermore, *Yu Ping Feng San (Chinese), Jade Windscreen Powder (English), Pulvis Paraventi Jaspidis (Latin)* from *Shi Yi De Xiao Fang* (Effective Formulas from Generations of Physicians) [110] composed of *Astragali membranacei radix, Atractylodis macrocephalae radix*, and *Saposhnikoviae radix*, traditionally considered as consolidating the body's immune defence, is present in this multiple decoction. 

This complex formula was investigated in the above-mentioned randomised, controlled, two-armed clinical trial on chemotherapy patients (*n* = 90; treatment: *n* = 50; control: *n* = 40) by oral administration to the treatment group [56]. In addition, several of their individual symptoms were considered in each case by adding herbal modules. In case of diarrhoea, neutropenia, thrombopenia, petechiae, lymphadenopathy, fever, insomnia, night sweat, or increased ministerial fire (which is a specific term of TCM theory), two different herbs were added to the recipe. 

This total of herbal medicine was compared to oral administration of vitamin B2 and vitamin C in the control group. All patients received dental care instructions and diet advices. Ulcer degree was classified by a subjective clinical scale. 

Though positive effects on mucositis symptoms were seen in 98% of the treatment group compared to 72% of the vitamin control group, the formula complexity does not allow congruent conclusions due to several variances in the treatment group. The multiple interactions between single herbal components do not allow a clear view on the (formula's) effects. Furthermore, vitamin application may be useful for oral mucositis patients but does not represent a valid control to any complex herbal prescription of this size.

## 6. Discussion

Chemotherapy-induced oral mucositis continues to be a challenge for anticancer treatment [8, 9, 12–15], representing one of the most common problems for chemotherapy patients [1]. Despite of the dedicated research on this field [1–7] and some resulting guidelines for prevention and treatment of oral mucositis [9, 10, 16, 17], therapeutic results are not yet satisfactory. Chemotherapy side effects in general represent an additional physical and psychological burden to patients diagnosed with cancer, reducing their quality of life and leading to the risk of anticancer treatment delay with fatal consequences [7, 8]. On the other hand, posttraumatic stress reactions as seen in cancer patients lead to a decreased defence against oral mucositis, determined by the alteration of several biomarkers [6, 7].

Medicinal herbs are commonly used for complementary treatment when there is no sufficient western treatment concept. A Chinese review identified diverse approaches regarding oral mucositis by applying herbal medicine, including various gargling preparations, sprays, formulas for oral administration, acupoint application or intravenous injection, resulting in a generally positive effect [111], but the evidence of herbal use on oral mucositis still remains unclear. 

Generally, systematic review studies on Chinese herbal medicine come to the conclusion that better qualified studies are necessary [112–114]. At the clinical level, study designs used to be mostly suboptimal but even on pharmacological level, the study quality is criticized as being sufficient. This is not surprising, because classical pharmacological research is generally focusing on single active compounds and this method of approach is not easily transferred to the multidimensional complexity of Asian herbal prescriptions. But in some aspects this view is short sighted, as a single-target approach can have limited effectiveness, and there is some evidence that a multi-target approach might be more effective [115, 116] and mixtures may have potentiating actions of their multiple bioactive components [117].

In this review, on the one hand we tried to summarize the state of knowledge of Chinese herbal treatment for chemotherapy-induced oral mucositis based on clinical trials. On the other hand, we tried to examine the TCM tradition based rationality of the particular herbs used for mucositis. 

While aggressive treatments like chemotherapy have not been used in the history of TCM, application of traditional Chinese herbal treatment to these side effects of modern therapy requires an intentional transfer of historical concepts to modern treatment procedures. In daily practice, Chinese herbal medicine has an individualized approach that cannot be easily transferred into standardized controlled trials due to the uniform treatment concepts usually required by controlled trials.

For a full understanding of the mechanisms of herbal prescriptions, the effects of every single herb must be known on a molecular basis. Based on these data, herbal combinations should be investigated for detecting synergisms that may result in molecular effects which are not found in the single herb components [118, 119]. Further elaborated research is necessary for clearing multiple questions about single as well as combined herbal use, resulting in the aim of rational prescription rather than application based only on empirical knowledge. 

Even though all reviewed clinical trials reported positive effects of Chinese herbal treatment, they did not show adequate study designs proportionately with regard to the investigated questions. Some studies used complex multi-herbal formulas that lead to difficulties in understanding the detailed effects. Formula complexity should be well elaborated in order to achieve significant results. Monoherbal applications offer clear results on the basis of well elaborated study designs. Herbal combinations may be even more effective in clinical results [115–117], while under clinical study conditions the evidence of exaggerated multi-herbal application is narrowed by numerous interferences between the single compounds, especially in combined prescriptions of variant formulas (see 5.2, 5.10, 5.11, 5.12, and 5.13).

The use of individualized herbal combinations has a long tradition in TCM on an empirical basis. One of the evaluated trials tries to take this classical approach into account by additional prescriptions depending on the accompanying symptomatology, resulting in extremely complex treatment procedures (see 5.13). In consequence, the general overview gets lost. In other trials treatment procedures of topical application and internal or even intravenous administration were combined (see 5.1a, 5.3, 5.6, and 5.10 and 5.11), making it impossible to draw applicable conclusions about one of the interventions. One trial used a formula mixed with vitamins (see 5.12), while others used vitamin B2, B12, and/or vitamin C application for controls without discussing the rationale (see 5.3, 5.6, 5.11, 5.12, and 5.13). In the same manner, it is difficult to judge the efficacy on herbal ice application versus ambient tempered gargle solution (see 5.4). Antibiotics were used for control groups in some studies (see 4.3, 5.6, and 5.11) while basic use of antibiotics challenged the result of one study (see 5.7).

Only in one study a non-treatment group had been established (see 4.2) and in only 6 studies the same type of application was used for treatment as well as for control groups (see 4.3, 4.4, 5.1b, 5.2, 5.5, and 5.7). Two authors reported uncontrolled case studies (see 4.1, 5.8, and 5.9).

In summary it is almost impossible to evaluate which parts of the treatment concepts are responsible for the measured effects in the reviewed trials. 

Another problem for judging study results is the fact that oral mucositis is a severe but short term side effect of chemotherapy, typically self-limiting in about 2 to 4 weeks if not complicated by infections [1]. Study designs have to consider that even without any specific treatments, symptoms possibly improve in this time. 

In general, the investigated trials showed low-quality designs. Control groups were established in most investigated trials but control interventions did not represent any standards. It has to be admitted that the mucositis guidelines so far existing are limited, so it is not easy to find valid control interventions for some treatment concepts. But in general, clinical studies should offer a standard basic treatment for all patients or placebo non-treatment groups in order to gather valid data on the investigated intervention. In the case of mucositis, establishing non-treatment groups could cause an ethical dilemma for having possible severe consequences [9, 12, 13] but it is possible to establish basic treatment conditions for all patients taking part of the study which do not interfere with the investigated intervention. This approach includes placebo administration for control groups in order to achieve valid data. In the case of Chinese medicinal herbs, the use of capsules containing herbal extractions or placebo is a good option. In the case of gargling solutions, fabricating a valid placebo may be more complicated but should not be impossible. Patients and medical practitioners should be blinded regarding the applied intervention in order to minimize placebo effects. The blinding technique has not been reported in any of the investigated studies. Randomisation has been reported in all controlled studies though the process has not been described except very briefly in two publications (5.1b and 5.12). For proving a strict randomisation protocol so as not to create bias, it is necessary to provide a detailed description.

Summarizing the collected data so far, results of Chinese medicinal herbal administration for chemotherapy-induced oral mucositis are potentially promising, but poor study designs do not allow valid conclusions. Conducting a meta-analysis is not possible with the present database. Further investigations are necessary on molecular mechanisms of multi-herbal formulas and the corresponding single herbs as well as in well designed clinical trials. Providing adequate study designs are developed, traditional Chinese herbal medicine has the potential of complementing western regimens such as chemotherapy in order to achieve lower levels of side effects, thus enabling patients to better resist chemotherapy impacts. 

## 7. Conclusion

All evaluated trials in this review reported positive effects about Chinese herbal treatment for chemotherapy-induced oral mucositis, but the value of these treatments remains unclear. Study designs are generally poor, some herbal prescriptions are far too complex and adequate controls are missing. Mechanisms of action are rarely described.

While basic research provides data about anti-inflammatory and protective effects of some herbs or herbal compounds, further research is still promising, but study designs need considerable improvement. So future research should start with mechanism based studies first. The following clinical studies should reduce the complexity of the treatment procedures in order to produce clear results, before Chinese herbal medicine can become an evidenced based part of the treatment of chemotherapy induced mucositis.

## Figures and Tables

**Table 1:** WHO oral toxicity scale [14, 15].

Grade 0	Grade I	Grade II	Grade III	Grade IV
(None)	(Mild)	(Moderate)	(Severe)	(Life-threatening)
None	Oral soreness, erythema	Oral erythema, ulcers, solid diet tolerated	Oral ulcers, liquid diet only	Oral alimentation impossible

**Table 2:** Clinical trials for chemotherapy-induced mucositis.

No.	Author/year	Formula	Drug application	Study design	Ulcer classification	Control group	Random/blinding	Effect/control, case number
4.1	Xu and Han2006 [28]	*Evodiae fr. *	Acupoint application	Case study	No	No	No/No	92% improved/no control *N* = 50

4.2	Loo et al. 2010 [31]	*Rhodiola algida *	Oral administration	Randomized group comparison	WHO scale	Basic treatment	Yes *5/No	Improved (ulcer size and number) *N* = 130

4.3	Shi and Shan 2009 [34]	*Catechu *	Topical application	Randomized group comparison	Clinical judgement	Topic norfloxacin powder	Yes *5/No	100%/73.3% improved *N* = 60

4.4	Wang et al. 2010 [37]	*Periplaneta americana *	Gargle solution	Randomized group comparison	WHO scale	Saline gargle	Yes *5/No	64%/16% improved *N* = 64

5.1a	Ma and Song 2005 [44]	*Lonicerae fl. Glycyrrhiza r. *	Oral administr. + gargle solution	Randomized group comparison	WHO scale	Boxax gargle	Yes *5/No	80.6%/14.8% improved *N* = 190

5.1b	Zeng 2005 [45]	*Lonicerae fl, Glycyrrhiza r. *	Gargle solution	Randomized group comparison	Clinical judgement	Peroxide gargle	Random number table/No	95.3%/76.7% improved *N* = 86

5.2	Bao et al. 2008 [47]	*Lonicerae fl. Ophiopogonis r. Astragali memb. r. *	Gargle solution	Randomized group comparison	WHO scale	Dobell's solution gargle	Yes *5/No	30%/5% improved for ulcer grading, no effect on healing time *N* = 97

5.3	Chen et al. 2005 [48]	*Lonicerae fl. Ophiopogonis r. Platycodonis r. *	Oral administration	Randomized group comparison	Ulcer size	Furacilin gargle oral vit B1 + C metronidazol infusion	Yes *5/No	93%/73.8% improved *N* = 65

5.4	Wu Zhu YF et al. 2009 [49]	*Lonicera fl. Glycyrrhiza r. Menthae hapl. h. Chrysanthemi fl. Ganoderma luc. *	Topical ice cube application	Randomized group comparison	WHO scale	Dobell's solution gargle	Yes *5/No	86.9%/60.9% improved *N* = 217

5.5	Wang et al. 2006 [50]	Sheng Mai San *1	Intravenous injection	Randomized group comparison	Clinical judgement	Dexamethasone MCP ondansetr. injection	Yes *5/No	94.5%/71.5% improved *N* = 71

5.6	Jin et al. 2009 [51]	*Bubali cornu, Callicarpae mac. fol. *	Oral administer. + gargle solution	Randomized group comparison	WHO scale VAS QOL	Saline, gentamycin, tetrahydrofolate gargle/oral oryzanol	Yes *5/No	93.3%/93.3%/7.8% improved *N* = 88

5.7	Hou et al. 2001 [52]	*Phellodendri c. Forsythiae fr. Galla chin. Verbenae h. Catechu* Borneolum + saline	Gargle solution	Randomized group comparison	Clinical judgement	No	Yes *5/No	96.2%/76.1% improved *N* = 101

5.8/5.9	Zhu and Zhang 1993 [53]	Yu Nu San*2/Qing Wei San*3	Oral administr.	Case study, clinical allocation to group 1 or 2	Clinical judgement	No	No/No	83.9 improved/no control *N* = 31

5.10/5.11	Zhou et al. 2005 [54]	*Chrysanthemi fl. Gardeniae fr Hypericum perf. Scrophulariae r. Sophora tonk. r./Borax, Borneolum, Indigo naturalis *	Oral administration + gargle solution	Randomized group comparison	Ulcer size, quantity	Vit B12, gentamicin and sodium bicarbonate gargle	Yes *5/No	96.7% improved, shorter healing period/86.7% improved *N* = 60

5.12	Chen and Zheng 2005 [55]	*Codonopsitis r. Atractylodis mac. rh. Agastachis h. Glycyrrhiza r. Dioscoreae rh Astragali memb. r. Angelica sin. r. Alismatis rh. Lopatheri h. Rehmannia vir. r. + Centrum Wyeth *	Oral administr. + gargle solution	Randomized group comparison	WHO scale	Vit B2	Envelope lottery/No	78.8%/33.3% improved *N* = 66

5.13	Sun 2007 [56]	Modified Xie Huang San*4 + added modules	Oral administr.	Randomized group comparison	WHO scale	Vit B2 and Vit C	Yes *5/No	98%/72% improved *N* = 90

*1: *Ginseng radix, Ophiopogonis radix, Schisandrae chinensis fructus*.

*2: *Gypsum fibrosum, Rehmanniae viridae radix, Anemarrhenae rhizoma, Ophiopogonis radix, Achyranthis bidentatae radix*.

*3: *Coptidis rhizoma, Cimicifugae rhizoma, Rehmanniae viridae radix, Moutan cortex, Angelicae sinensis radix, Achyranthis bidentatae radix*.

*4: *Gypsum fibrosum, Saposhnikoviae radix, Gardeniae fructus, Agastachis herba, Glycyrrhizae radix, Astragali membranacei radix, Atractylodis macrocephalae rhizoma, Dendrobii herba, Lophatheri herba, Hedyotidis herba, Taraxaci herba, Coptidis rhizoma, Ginseng radix*, plus added modules.

*5: Randomisation process not described.

## References

[1] http://www.cancer.gov/cancertopics/pdq.

[2] Rostock M, Saller R (2007). Schleimhautschäden unter antitumoraler Behandlung: Präventive und therapeutische Möglichkeiten mit pflanzlichen Zubereitungen. *Schweizerische Zeitschrift für Ganzheitsmedizin*.

[3] Epstein JB, Tsang AHF, Warkentin D, Ship JA (2002). The role of salivary function in modulating chemotherapy-induced oropharyngeal mucositis: a review of the literature. *Oral Surgery, Oral Medicine, Oral Pathology, Oral Radiology, and Endodontics*.

[4] Scully C, Epstein J, Sonis S (2003). Oral mucositis: a challenging complication of radiotherapy, chemotherapy, and radiochemotherapy. Part 1, pathogenesis and prophylaxis of mucositis. *Head and Neck*.

[5] Peterson DE, Lalla RV (2010). Oral mucositis: the new paradigms. *Current Opinion in Oncology*.

[6] Xu C, Zhao J, Loo WT (2010). Correlation of epigenetic change and identification of risk factors for oral submucous fibrosis. *International Journal of Biological Markers*.

[7] Bai LJ, Liu Q, Wang M (2012). Evaluation of the psychological and biological changes of patients diagnosed with benign and malignant breasttumors. *International Journal of Biological Markers*.

[8] McGowan D (2008). Chemotherapy-induced oral dysfunction: a literature review. *British Journal of Nursing*.

[9] Wojtaszek C (2000). Management of chemotherapy-induced stomatitis. *Clinical Journal of Oncology Nursing*.

[10] Keefe DM, Schubert MM, Elting LS (2007). Updated clinical practice guidelines for the prevention and treatment of mucositis. *Cancer*.

[11] Mucositis Study Section of the Multinational Association of Supportive Care in Cancer Mucositis, perspectives and clinical practice guidelines, perspectives on cancer therapx-induced mucosal injury. http://www3.interscience.wiley.com/cgi-bin/fulltext/108069519.

[12] Spijkervet FKL, Sonis ST (1998). New frontiers in the management of chemotherapy-induced mucositis. *Current Opinion in Oncology*.

[13] Khan SA, Wingard JR (2001). Infection and mucosal injury in cancer treatment. *Journal of the National Cancer Institute. Monographs*.

[14] (1979). *WHO Handbook*.

[15] Sonis ST, Elting LS, Keefe D (2004). Perspectives on cancer therapy-induced mucosal injury. *Cancer*.

[16] Lalla RV, Sonis ST, Peterson DE (2008). Management of oral mucositis in patients with cancer. *Dental Clinics of North America*.

[17] http://www.mascc.org/mucositis-guidelines.

[18] Worthington HV, Clarkson JE, Bryan G (2010). Interventions for preventing oral mucositis for patients with cancer receiving treatment. *Cochrane Database of Systematic Reviews*.

[19] Worthington HV, Clarkson JE, Bryan G (2011). Interventions for preventing oral mucositis for patients with cancer receiving treatment. *Cochrane Database of Systematic Reviews*.

[20] Ernst E, Cassileth BR (1998). The prevalence of complementary/alternative medicine in cancer: a systematic review. *Cancer*.

[21] Boon H, Brown JB, Gavin A, Kennard MA, Stewart M (1999). Breast cancer survivors’ perceptions of complementary/alternative medicine (CAM): making the decision to use or not to use. *Qualitative Health Research*.

[22] Tindle HA, Davis RB, Phillips RS, Eisenberg DM (2005). Trends in use of complementary and alternative medicine by us adults: 1997–2002. *Alternative Therapies in Health and Medicine*.

[23] Chan CWH, Chang AM, Molassiotis A, Lee IYM, Lee GCT (2003). Oral complications in Chinese cancer patients undergoing chemotherapy. *Supportive Care in Cancer*.

[24] Porkert M (1994). *Klinische Chinesische Pharmakologie*.

[25] Bensky D, Clavey S, Stoger E (2004). *Chinese Herbal Medicine: Materia Medica*.

[26] Chinese Pharmacopoeia Commision (2010). *Pharmakocoeia of the People' Republic of China*.

[27] Yu H, Jin H, Gong W, Wang Z, Liang H (2013). Pharmacological actions of multi-target-directed evodiamine. *Molecules*.

[28] Xu TL, Han JH (2006). External application of Evodia rutaecarpa on Acupoint Yongquan in remaining dental ulcer after chemotherapy. Zhu Yu waifu yong quam xue ke zhiliao hualiao hou kouqiang kui yang. *Chinese Journal of Nursing*.

[29] http://www.shen-nong.com/eng/herbal/hongjingtian.html.

[30] Li HX, Sze SCW, Tong Y, Ng TB (2009). Production of Th1- and Th2-dependent cytokines induced by the Chinese medicine herb, Rhodiola algida, on human peripheral blood monocytes. *Journal of Ethnopharmacology*.

[31] Loo WTY, Jin L, Chow LWC, Cheung MNB, Wang M (2010). Rhodiola algida improves chemotherapy-induced oral mucositis in breast cancer patients. *Expert Opinion on Investigational Drugs*.

[32] http://www.naturalremedy.com/Acacia%20catechu.pdf.

[33] Tseng-Crank J, Sung S, Jia Q (2010). A medicinal plant extract of scutellaria baicalensis and acacia catechu reduced LPS-stimulated gene expression in immune cells: a comprehensive genomic study using QPCR, ELISA, and microarray. *Journal of Dietary Supplements*.

[34] Shi YJ, Shan JZ (2009). Observation on the effect of catechin from traditional Chinese medicine on oral ulcer induced by chemotherapy. *Journal of Clinical Nursing*.

[35] Chen HX, Liang XH, Jiang LH (2011). Clinical effect of Kangfuxin liquid in treatment of recurrent aphtous ulcer. *China Practical Medicine*.

[36] Wang LM, Lu YM, Yu J, Zhu DS, Chen WX (2006). Inhibition of the expression of cytokines on experimental colitis in mice by kangfuxin. *Chinese Journal of Clinical Pharmacology*.

[37] Wang H, Xu XF, Zeng ZW (2010). Rehabilitation drug solution on prevention and treatment of chemotherapy-induced oral ulcers. *Medical Information*.

[38] Wu JT (2005). *Wen Bing Tiao Bian (Systematic Differentiation of Warm Diseases)*.

[39] Scheid V, Bensky D, Ellis A, Barolet R (2004). *Chinese Herbal Medicine: Formulas and Strategies*.

[40] Porkert M (1994). *Klassische Chinesische Rezeptur*.

[41] Liao Y, Dong S, Kijama R, Cai P, Liu L, Shen H (2013). Flos lonicerae exracts and chlorogenic acid protect human umbilical vein endothelia cells from the toxic damage of perfluorooctane sulphonate. *Inflammation*.

[42] Shin EM, Zhou HY, Guo LY (2008). Anti-inflammatory effects of glycyrol isolated from Glycyrrhiza uralensis in LPS-stimulated RAW264.7 macrophages. *International Immunopharmacology*.

[43] Kim YW, Zhao RJ, Park SJ (2008). Anti-inflammatory effects of liquiritigenin as a consequence of the inhibition of NF-*κ*B-dependent iNOS and proinflammatory cytokines production. *British Journal of Pharmacology*.

[44] Ma ZQ, Song XH (2005). Licorice and Honeysuckle decoction for prevention oral ulcer by high dose chemotherapy: a clinical observation. Gan cao Jin Hua jian yin yufang da ji liang hualiao kouqiang kuiyang de linchuang guancha. *Modern Journal of Lntegrated Traditional Chinese and Western Medicine*.

[45] Zeng YX (2005). Yin Hua Gan Cao decoction was used to treat stomatitis caused by chemical therapy. Yin Hua Gan Cao Tang yongyu hualiao bingren kouqiang yan de huli tihui. *Guiding Journal of TCM*.

[46] Nalbantsoy A, Nesil T, Yilmaz-Dilsiz O, Aksu G, Khan S, Bedir E (2012). Evaluation of the immunomodulatory properties in mice and in vitro anti-inflammatory activity of cycloartane type saponins from Astragalus species. *Journal of Ethnopharmacology*.

[47] Bao NX, Zeng GZ, Zhou LW, Shu XY, Yang L (2008). Preventive effect of self-made gargle solution on chemotherapy-induced oral ulcer in patients with colorectal carcinoma. *Journal of Nursing Science*.

[48] Chen JY, Peng AL, Hao GZ, Zhu H, Yao YZ (2005). Curative effect of ophiopogonon tuber mixture on ulcer of oral cavity after chemotherapy. Mai Dong He Ji zhiliao hualiao hou kouqian kuiyang xiaoguo guancha. *Journal of Nursing Science*.

[49] Wu Zhu YF XE, Lai YM, Xie XH (2009). Gargle medicine ice observation on the effects of chemotherapy in patients with oral ulcer. *Journal of Nursing*.

[50] Wang LH, Dai AW, Wang L (2006). The effect of Shengmai injection on alleviating acute chemotherapy toxicity. Wu Zhu Yu waifu yongquan xue ke zhiliao hualiao hou kouqian kuiyang. *New Journal of Traditional Chinese Medicine*.

[51] Jin T, Shen MH, Sun YF, Zhang J (2009). Water Grass Decoction for treating Chemotherapy-induced Oral Ulcers. Shui Zhong Cao Tang Ji zhiliao hualiao suozhi kouqiang kuiyang. *Chinese Arcieves of Raditional Chinese Medicine*.

[52] Hou FJ, Jin BC, Li W (2001). Observation on the effects of yellow five gargle for oral ulcers caused by chemotherapy. Huang Wu shukouye yongyu hualiao suoshi kouqiang kuiyang de xiaoguo guancha. *Journal of Nursing Science*.

[53] Zhu H, Zhang J (1993). Treatment of stomatological complications in 31 cases of acute leukemia with Chinese herbal drugs. *Journal of Traditional Chinese Medicine*.

[54] Zhou ZX, Zhang ZR, Qian HX, Li ZQ, Wu WM, Luo J (2005). Traditional Chinese medicine in preventing and treating oral ulcer induced by chemotherapy clinical observation. *Journal of Sichuan of Traditional Chinese Medicine*.

[55] Chen CY, Zheng YZ (2005). Clinical study on prevention of oral ulcers caused by chemotherapy with the combined therapy of centrum and specific Chinese herbs that invigorate the function of the spleen and regulate the flow of Qi. *Chinese Journal of Clinical Nutrition*.

[56] Sun JH (2007). Clinical Observation of modified Xiehuang Powder in the Treatment of Oral Ulcer following Chemotherapy. Xie Huang San jiawei zhiliao hualiao hou kouqiang kuiyang linchuang guancha. *Modern Journal of Integrated Traditional Chinese and Western Medicine*.

[57] Wang A (2006). *Yi Fang Yi Jie (Analytic Collection of Medical Formulas)*.

[58] Wu X, Dai H, Huang L, Gao X, Tsim KWK, Tu P (2006). A fructan, from radix ophiopogonis, stimulates the proliferation of cultured lymphocytes: structural and functional analyses. *Journal of Natural Products*.

[59] Kim JY, Hwang YP, Kim DH (2006). Inhibitory effect of the saponins derived from roots of Platycodon grandiflorum on carrageenan-induced inflammation. *Bioscience, Biotechnology and Biochemistry*.

[60] Cheon MS, Yoon T, Lee DY (2009). Chrysanthemum indicum Linné extract inhibits the inflammatory response by suppressing NF-*κ*B and MAPKs activation in lipopolysaccharide-induced RAW 264.7 macrophages. *Journal of Ethnopharmacology*.

[61] Sun L-X, Chen L-H, Lin Z-B (2011). Effects of Ganoderma lucidum polysaccharides on IEC-6 cell proliferation, migration and morphology of differentiation benefiting intestinal epithelium healing in vitro. *Journal of Pharmacy and Pharmacology*.

[62] Tie L, Yang H-Q, An Y (2012). Ganoderma lucidum polysaccharide accelerates refractory wound healing by inhibition of mitochondrial oxidative stress in type 1 diabetes. *Cellular Physiology and Biochemistry*.

[63] Yoon HM, Jang KJ, Han MS (2013). Ganoderma lucidum ethanol extract inhibits the inflammatory response by suppressing the NF-kB and toll-like receptor pathways in lipopolysaccharide-stimulated BV2 microglial cells. *Experimental and Therapeutic Medicine*.

[64] Wong RWK, Hägg U, Samaranayake L, Yuen MKZ, Seneviratne CJ, Kao R (2010). Antimicrobial activity of Chinese medicine herbs against common bacteria in oral biofilm. A pilot study. *International Journal of Oral and Maxillofacial Surgery*.

[65] Chan BCL, Hon KLE, Leung PC (2008). Traditional Chinese medicine for atopic eczema: penta herbs formula suppresses inflammatory mediators release from mast cells. *Journal of Ethnopharmacology*.

[66] Migliorati CA, Oberle-Edwards L, Schubert M (2006). The role of alternative and natural agents, cryotherapy, and/or laser for management of alimentary mucositis. *Supportive Care in Cancer*.

[67] Zhang YS (2002). *Yu Xue Qi Yuan (Expounding on the Origins of Medicine): Yuan Dynasty*.

[68] Lee DCW, Lau ASY (2011). Effects of Panax ginseng on tumor necrosis factor-*α*-mediated inflammation: a mini-review. *Molecules*.

[69] Kim WK, Song SY, Oh WK (2013). Wound-healing effect of ginsenoside Rd from leaves of Panax ginseng via cyclic AMP-dependent protein kinase pathway. *European Journal of Pharmacology*.

[70] Nishida H, Tatewaki N, Nakajima Y (2009). Inhibition of ATR protein kinase activity by schisandrin B in DNA damage response. *Nucleic Acids Research*.

[71] Takimoto Y, Qian HY, Yoshigai E, Okomura T, Ikeya Y, Nishizawa M (2013). Gomisin N in the herbal drug gomishi (Schisandra chinensis) suppresses inductible nitric oxide synthase gene via C/EBPbeta and NF-kB in rat hepatocytes. *Nitric Oxide*.

[72] Liu R, Wang M, Duan J-A (2010). Antipyretic and antioxidant activities of the aqueous extract of Cornu Bubali (water buffalo horn). *The American Journal of Chinese Medicine*.

[73] Tu Y, Sun L, Guo M, Chen W (2013). The medicinal uses of Callicarpa L. in traditional Chinese medicine: an Ethnopharmacological, phytochemical and pharmacological review. *Journal of Ethnopharmacology*.

[74] Li DY (2005). *Lan Shi Mi Cang (Secrets From the Orchid Chamber)*.

[75] Park Y-K, Chung YS, Kim YS, Kwon O-Y, Joh TH (2007). Inhibition of gene expression and production of iNOS and TNF-*α* in LPS-stimulated microglia by methanol extract of Phellodendri cortex. *International Immunopharmacology*.

[76] Kim K, Lee S, Shin S (2011). Anti-inflammatory function of arctiin by inhibiting COX-2 expression via NF-*κ*B pathways. *Journal of Inflammation*.

[77] http://www.epharmacognosy.com/2012/08/european-verbena-herb-ma-bian-cao.html.

[78] Speroni E, Cervellati R, Costa S (2007). Effects of differential extraction of Verbena officinalis on rat models of inflammation, cicatrization and gastric damage. *Planta Medica*.

[79] Liu R, Zhang L, Lan X (2011). Protection by borneol on cortical neurons against oxygen-glucose deprivation/reperfusion: involvement of anti-oxidation and anti-inflammation through nuclear transcription factor *κ*appaB signaling pathway. *Neuroscience*.

[80] Zhang JB (1997). *Collected Treatise of [Zhang] Jing-Yue: 1624. Jing Yue Quan Shu*.

[81] Wang HD, Wang MY, Li XB (2009). Evaluation of the antipyretic activity of Gypsum Fibrosum and its constituents. *Asian Journal of Traditional Medicines*.

[82] Liu CL, Cheng Ko CH L, Wong CW (2012). Bioassay-guided isolation of anti-inflammatory components from the root of Rehmannia glutinosa and ist underlying mechanism via inhibition of iNOS pathway. *Journal of Ethnopharmacology*.

[83] Lee HJ, Li H, Chang HR, Jung H, Lee DY, Ryu JH (-)-Nyasol, isolated from Anemarrhena asphodeloides suppresses neuroinflammatory response through the inhibition of I-alphaBa degradation in LPS-stimulated BV-2 microglial cells.

[84] Zou Y, Meng J, Chen W (2011). Modulation of phenotypic and functional maturation of murine dendritic cells (DCs) by purified Achyranthes bidentata polysaccharide (ABP). *International Immunopharmacology*.

[85] Kim JM, Jung HA, Choi JS, Lee NG (2010). Identification of anti-inflammatory target genes of Rhizoma coptidis extract in lipopolysaccharide-stimulated RAW264.7 murine macrophage-like cells. *Journal of Ethnopharmacology*.

[86] Schmid D, Woehs F, Svoboda M, Thalhammer T, Chiba P, Moeslinger T (2009). Aqueous extracts of Cimicifuga racemosa and phenolcarboxylic constituents inhibit production of proinflammatory cytokines in LPS-stimulated human whole blood. *Canadian Journal of Physiology and Pharmacology*.

[87] Yun CS, Choi YG, Jeong MY, Lee JH, Lim S (2012). Moutan cortex radicis inhibits inflammatory changes og gene expression in lipopolysaccharide-stimulated gingival fibroblasts. *Journal of Natural Medicines*.

[88] Lee HJ, Li H, Chang HR, Jung H, Lee DY, Ryu JH (2013). (-)-Nyasol, 10 isolated from Anemarrhena asphodeloides suppresses neuroinflammatory response through the inhibition of I-alphaBa degradation in LPS-stimulated BV-2 microglial cells. *Journal of Enzyme Inhibition and Medicinal Chemistry*.

[89] Fu Y, Liu B, Liu J (2012). Geniposide, from Gardenia jasminoides Ellis, inhibits the inflammatory response in the primary mouse macrophages and mouse models. *International Immunopharmacology*.

[90] Huang N, Rizshsky L, Hauck CC, Nikolau BJ, Murphy PA, Birt DF (2012). The inhibition of lipopolysaccharide-induced macrophage inflammation by 4 compounds in Hypericum perforatum extract is partially dependent on the activation of SOCS3. *Phytochemistry*.

[91] Hempen CH, Fischer T (2006). *Leitfaden Chinesische Phytotherapie*.

[92] Guo J-P, Pang J, Wang X-W, Shen Z-Q, Jin M, Li J-W (2006). In vitro screening of traditionally used medicinal plants in China against enteroviruses. *World Journal of Gastroenterology*.

[93] Wu Q (2006). *Yi Zong Jin Jian (Golden Mirror of the Medical Tradition)*.

[94] Turkez H, Geyikoglu F, Tatar A, Keles MS, Kaplan I (2012). The effects of some boron compounds against heavy metal toxicity in human blood. *Experimental and Toxicologic Pathology*.

[95] Pongsavee M (2009). Effect of borax on immune cell proliferation and sister chromatid exchange in human chromosomes. *Journal of Occupational Medicine and Toxicology*.

[96] Lin Y-K, Leu Y-L, Huang T-H (2009). Anti-inflammatory effects of the extract of indigo naturalis in human neutrophils. *Journal of Ethnopharmacology*.

[97] Xue J (1995). *Zheng Ti Lei Yao (Catalogued Essentials For Correcting the Body)*.

[98] Tai Ping Hui Min He Ji Ju

[99] Li DY (2007). *Nei Wai Shang Bian Huo Lun (Clearing Doubts About Injury From Internal and External Causes)*.

[100] http://www.tcmassistant.com.

[101] Lee YG, Kim JY, Lee JY (2007). Regulatory effects of Codonopsis lanceolata on macrophage-mediated immune responses. *Journal of Ethnopharmacology*.

[102] Kim S, Shin S, Hyun B (2012). Immunmodulatory effects of dioscoreae rhizome against inflammation through suppressed production of cytokines via inhibition of the NF-kappaB pathway. *Immune Network*.

[103] Han CW, Kwun MJ, Kim KH (2013). Ethanol extract of alismatis rhizoma reduces acute lung inflammation by suppressing NF-kappaB and activating Nrf2. *Journal of Ethnopharmacology*.

[104] Oh HM, Kang YJ, Lee YS (2006). Protein kinase G-dependent heme oxygenase-1 induction by Agastache rugosa leaf extract protects RAW264.7 cells from hydrogen peroxide-induced injury. *Journal of Ethnopharmacology*.

[105] Wang Y, Chen M, Zhang J (2012). Flavone C-glycosides from the leaves of Lophatherum gracile and their in vitro antiviral activity. *Planta Medica*.

[106] Qian Y (2007). *Xiao Er Yao Zheng Zhi Jue (Craft of Medicines and Patterns For Children)*.

[107] Tai J, Cheung S (2007). Anti-proliferative and antioxidant activities of Saposhnikovia divaricata. *Oncology Reports*.

[108] Zhang X, Xiong H, Liu L (2012). Effects of taraxasterol on inflammatory responses in lipopolysaccharide- induced RAW 264.7 macrophages. *Journal of Ethnopharmacology*.

[109] Lin X, Shaw P-C, Sze SC-W, Tong Y, Zhang Y (2011). Dendrobium officinale polysaccharides ameliorate the abnormality of aquaporin 5, pro-inflammatory cytokines and inhibit apoptosis in the experimental Sjögren’s syndrome mice. *International Immunopharmacology*.

[110] Wei YL (2006). *Shi Yi De Xiao Fang (Effective Formulas From Generations of Physicians)*.

[111] Wang CL (2011). Present situation of study on TCM prevention and treatment of oral ulcer induced by chemotherapy. *Journal of Nursing*.

[112] Liu JP, Zhang M, Wang WY, Grimsgaard S (2004). Chinese herbal medicines for type 2 diabetes mellitus. *Cochrane Database of Systematic Reviews*.

[113] Cho WCS (2010). Scientific evidence on the supportive cancer care with chinese medicine. *Chinese Journal of Lung Cancer*.

[114] Li SG, Chen HY, Ou-Yang CS (2013). The efficacy of Chinese herbal medicine as an adjunctive therapy, for advanced non-small cell lung cancer: a systematic review and meta-analysis. *PLoS ONE*.

[115] Tian X-Y, Liu L (2012). Drug discovery enters a new era with multi-target intervention strategy. *Chinese Journal of Integrative Medicine*.

[116] Lu J-J, Pan W, Hu Y-J, Wang Y-T (2012). Multi-target drugs: the trend of drug research and development. *PLoS ONE*.

[117] Schmidt BM, Ribnicky DM, Lipsky PE, Raskin I (2007). Revisiting the ancient concept of botanical therapeutics. *Nature Chemical Biology*.

[118] Wu L, Ding XP, Zhu DN, Yu BY, Yan YQ (2010). Study on the radical scavengers in the traditional Chinese medicine formula Shengmai San by HPLC-DAD coupled with chemiluminescence (CL) and ESI-MS/MS. *Journal of Pharmaceutical and Biomedical Analysis*.

[119] Suzuki Y, Goto K, Ishige A, Komatsu Y, Kamei J (1999). Antinociceptive effect of Gosha-jinki-gan, a Kampo medicine, in streptozotocin-induced diabetic mice. *Japanese Journal of Pharmacology*.

